# Ascending Paralysis after Whooping-Cough

**Published:** 1887-10

**Authors:** 


### ASCENDING PARALYSIS AFTER WHOOPING-COUGH.

(Professor Moebius, *Centralblatt f. Nervenheilkunde*, 5, 1887.)

Moebius reports a case from his polyclinic wherein a boy of three years during the acme of whooping-cough developed a paresis, first of the lower extremities, then of the arms, the cervical muscles, and of the diaphragm. The tendon-reflexes were absent, but disturbances of sensibility of the superficial reflexes, of the functions of the cerebral nerves, and of the intestinal canal could not be demonstrated. The lesion, limited to the motory sphere, could not be a severe one, as atrophy and changes in the electrical irritability were absent, and because full restoration followed in a short time. The ascending paresis was not a steady one, and left abdominal and intercostal muscles, as well as those of the back, untouched; death, otherwise, would have been inevitable, as simultaneous palsy of the intercostal muscles and of the diaphragm, even if only existing for a little time, necessarily leads to a fatal result. Which parts of the motory apparatus were affected is a question of secondary importance; it might have been a slight myelitis or a so-called multiple neuritis, and the latter supposition is the more probable one, as neither micturition nor defecation was ever interfered with, and the disease ran a rapid course.

The chief question remains, what relation exists between the paralysis and the preceding infectious disease, the whooping-cough? and we may consider it a nervous sequela, analogous to the diphtheritic paralysis. We may suppose that in every acute infectious disease a peculiar poison is developed, which, according to its affinity to certain nerve fibres, causes a peculiar nervous affection, as we know already from the diphtheritic poison, and it seems as if most morbid noxæ exert a more diffuse action, injuring one or another section or sections of the nervous system, and it needs yet many an observation to differentiate between the nervous sequelæ of the acute infectious diseases, as whether an ascending paralysis is characteristic for whooping-cough. Our literature is very poor yet on similar observations. Most cases rather show focal diseases in the brain. Two cases observed by Moebius, as sequelæ of whooping-cough, were rather the one a cerebral infantile paralysis, and the other a poliomyelitis acuta. Ferber observed an acute mental alienation after whooping-cough. Cazin reports a case of a child suffering from moderate whooping-cough, but remarkably delicate and apathetic, who was taken with severe convulsions of the right side, and died in half an hour. Considerable hemorrhage between cranium and dura was found in the posterior half of the left hemisphere, and Cidal and Danchez report similar hemorrhages in the brain after whooping-cough in the *Progrès Médical.* West reports in the *British Med. Journal* of January, 1887, a case of right-sided hemiphlegia with aphasia and lithetosis appearing during whooping-cough.

It seems that the treatment instituted at the Berlin Polyclinic consisted in mild electrical treatment and massage, adjuvants which we ought not to neglect in similar cases, plus good, easily digestible food and rest. If I am not mistaken, a kind of Wier Mitchell treatment, individualized according to the nature of the case, would save many a patient who succumbed to a weakened heart after diphtheria, and the same may happen in whooping-cough. But we have many drugs which may aid us in restoring that disturbed nervous equilibrium, and none is here more neglected than that despised *Psorinum,* for we find among its symptoms: Paralytic debility without structural changes (hence in many cases the *vis medicatrix naturæ* suffices to brace up the nerves again, if aided by nutrition and rest); trembling of feet and hands; and Lippe considers it *the* remedy for the sequelæ of debilitating acute diseases. We always considered Psora as a deficiency of vital power, hence no resisting power to inimical noxæ, whether they come from outside or arise in our body, and Psorinum or any one of Hahnemann's antipsorica indicated. We just think of *Conium*, with its upward progressing paralysis; of *Cicuta*, that great remedy in cerebro-spinal meningitis; of Schüssler's *Kali phosphoricum* in paralysis dependent on exhaustion of nerve-power after recent infectious diseases; of *Manganum*, with its ascending multiple neuritis and consequent paresis, and especially of *Latyrus*, in Moebius' case, for it gives us: Paresis of lower extremities, with tremulous, tottering gait, sensibility remaining intact or even hyperæsthetic; no pains; worse when standing or walking, and better by lying down; whereas, in *Physostygma*, though it is more indicated as long as no structural lesions have taken place, we still think more of this drug in functional disturbances of the cord, purely spinal, with normal intellect. The very opposite of Latyrus we have in *Silicea*, where we also meet paralysis from defective nutrition of the nerves, but at the same time oversensitiveness to nervous stimuli and those wandering pains passing quickly from one part of the body to another. A weakened nervous system, momentarily relieved by alcoholic beverages or other stimulants (vin Mariani) hints to *Gelsemium*, and it has won many a blessing in post diphtheritic paralysis, with its tired, exhausted feeling of mind and body.

Kapka, in his classical work, on *Homœopathische Therapie*, mentions incidentally as a sequelæ of whooping-cough a partial or total paralysis of one or another extremity, and fears in all such cases a basilar meningitis on a tubercular foundation, and in other cases a tabes or marasmus sets in, based on an obstinate gastric catarrh. In all such developed cases the glandular system is suffering, nutrition suffers, and according to the suffering parts the symptoms and the treatment must vary. The cerebral hemorrhages are thus more easily explained, as a weakened vasomotor system relaxes the arterial walls and a break may follow, or there is a serous exudation, and in their debilitated state the prognosis becomes more than ominous.

Sulphur, Calcarea, Iod., Phosphor., and Arsenicum in their different combinations are the sheet-anchors in such threatening sequelæ, plus good nutrition, fresh air, and rest. According to Kapka, they are really our tonics, our roborantia, and it is just in such cases where Grauvogel's alternation of a high and low potency of the same remedy finds its suitable application—the low potency as the nutritive drug, the high potency in order to stop the functional derangement, so that it may not become organic, and when we look at the pathogenesis of our antipsoric remedies we will find them more frequently indicated during the different stages of this convulsive cough than is done by most physicians. Hence, perhaps, our frequent failures in shortening the disease, and it is well if we learn something by our failures.

Generalizing the pathological state and individualizing the drug suitable to the daily symptoms must go hand in hand, and success will crown the efforts of the painstaking physician; let us adhere closely to the dietary rules as found in the *Organon*—they ought never to be neglected—that exhausted nerve-power needs rest and passive exercise for its restoration, and our well-indicated remedy will hasten to bring back the bloom of health to our darling little sufferers. S. L.

